# Molecular detection of *Tritrichomonas foetus* in bovine samples: a novel real-time polymerase chain reaction (PCR) assay targeting *EF1-alpha-Tf1* and a comparative study of published PCR techniques

**DOI:** 10.1007/s00436-022-07487-7

**Published:** 2022-03-29

**Authors:** Coral Polo, Teresa García-Seco, Víctor Fernández, Marta Hernández, Victor Briones, Alberto Diez-Guerrier, Lucas Domínguez, Marta Pérez-Sancho

**Affiliations:** 1 VISAVET Health Surveillance Centre, Universidad Complutense de Madrid, Av. Puerta de Hierro s/n, 28040 Madrid, Spain; 2 MAEVA SERVET S.L., Calle de la Fragua 3, 28749 Madrid, Spain; 3 Zootecnia Análisis Clínicos Veterinarios S.L.P., Calle Tierra de Campos 24-26, 37008 Salamanca, Spain; 4 Molecular Biology and Microbiology Laboratory, Instituto Tecnológico Agrario de Castilla Y León, Av. Burgos, 47071 Valladolid, Spain; 5 Department of Animal Health, Faculty of Veterinary Science, Universidad Complutense de Madrid, Av. Puerta de Hierro s/n, 28040 Madrid, Spain

**Keywords:** *Tritrichomonas foetus*, Real-time PCR, *EF1-alpha-Tf1*, Bull

## Abstract

The parasite *T. foetus* causes trichomonosis in cattle but is generally asymptomatic in males. Thus, many bulls carrying the disease go unnoticed, making the detection of *T. foetus* in bulls an important aspect for its control. Due to drawbacks posed by its cultivation, PCR is a preferred option for diagnostic laboratories. Most published PCR protocols target the genomic region compring the *18S*, *5.8S*, and *28S* rRNA genes and internal transcribed spacers 1 and 2 (rRNA-ITS region), homologous to that of other *Tritrichomonas* species. There is minimal information on alternative genetic targets and no comparative studies have been published. We compared a protocol based on the microsatellite *TfRE* (called H94) and five protocols based on the rRNA-ITS region (called M06, M15, G02, G05, and N02). We also designed and evaluated a novel PCR-based assay on the *EF1-alpha-Tf1* gene (called V21). The analytical sensitivity and specificity assays for the PCR protocols were performed according to the World Organisation for Animal Health (OIE) directives and the comparative study was performed with a widely used PCR (M06) on clinical samples from 466 breeding bulls. V21 showed a high degree of agreement with our reference M06 (kappa = 0.967), as well as M15 (kappa = 0.958), G05 (kappa = 0.948), and H94 (kappa = 0.986). Protocols H94 and V21 appear to be good approaches for confirming clinical cases in preputial bull samples when genomic regions alternative to rRNA-ITS are required. By contrast, N02 gave false negatives and G02 false positives.

## Introduction

The protozoan *Tritrichomonas foetus* is a flagellated pathogen that causes trichomonosis in cattle, cats, and pigs (Dąbrowska et al. 2019b). In bulls, trichomonosis is an asymptomatic and persistent venereal disease, whereas in cows, it causes cervicitis, endometritis, foetal death during the first trimester of gestation, a delayed return to estrus, and infertility (Michi et al. 2016). In addition, *T. foetus* is transmitted through both natural breeding and artificial insemination (Givens 2018). In this scenario, the control of *T. foetus* in males is crucial for its control within cattle herds. Due to its relevance in production, bovine trichomonosis is a notifiable disease in certain countries.

The detection of *T. foetus* is generally carried out by microscopy, culture, and molecular techniques, such as PCR (OIE 2018). However, sampling and transport conditions can affect the detection rate of *T. foetus*, primarily via direct microscopy and culture (Yao 2013). In recent years, molecular detection has become one of the most widely used options in diagnostic laboratories (OIE 2018). The OIE cited real-time PCR, mentioning the work of McMillen and Lew (McMillen and Lew 2006) (here called the M06 protocol) as a suitable method for the detection of *T. foetus* in clinical samples (OIE 2018). This widely used PCR assay (Effinger et al. 2014; Dąbrowska et al. 2019a; Meggiolaro et al. 2019) is based on the genomic region comprising the *18S*, *5.8S*, and *28S* rRNA genes and internal transcribed spacers 1 and 2 (rRNA-ITS region), a multicopy target (Chakrabarti et al. 1992) used for the design of various published PCR assays (Felleisen et al. 1998; Gookin et al. 2002, 2005; Nickel et al. 2002; McMillen and Lew 2006; Mueller et al. 2015; Ginter Summarell et al. 2018; Dąbrowska et al. 2019a), most of them used for the diagnosis of clinical samples (Köster et al. 2015; Casteriano et al. 2016; Li et al. 2016; Dąbrowska et al. 2020). Despite the value of this diagnostic approach, to our knowledge, no systematic comparative study of all the PCR assays for the detection of *T. foetus* has been published. In addition, similarities with rRNA-ITS sequences present in other *Tritrichomonas* species, such as *Tritrichomonas mobilensis*, have been described, resulting in potential cross-reactions (Dąbrowska et al. 2019a). Alternative genetic regions have been studied (Reinmann et al. 2012; Šlapeta et al. 2012; Sun et al. 2012) for different purposes. Nevertheless, two alternatives PCR targets for the diagnosis of *T. foetus* directly in clinical samples, which may overcome these potential drawbacks, have been described: (i) *TfRE* microsatellite-PCR (Ho et al. 1994), with minimal information (only one reference sequence in GenBank (AY435432.1) that comes from an unpublished study), and (ii) a commercial kit that targets the *beta-tubulin 1* gene that lacks published data (OIE 2018). In this context, it would be useful to explore new molecular targets for the detection of *T. foetus* as a confirmatory test, for example, in the event of doubtful results. Oyhenart et al. recently published a novel loop-mediated isothermal amplification (LAMP) assay targeting the *EF1-alpha-Tf1* gene for the detection of *T. foetus* on bull preputial wash samples (Oyhenart 2018). This LAMP assay showed high analytical sensitivity (0.5 trophozoites/mL) and specificity, making this genetic target a valuable alternative for the confirmation of *T. foetus* in clinical samples (Oyhenart 2018). However, no PCR assay for *T. foetus* detection directly in clinical samples based on this target has yet been published.

Here, we aimed to perform a comparative study of all molecular target-PCR techniques currently published for the detection of *T. foetus* to assess their diagnostic performance on a cohort of breeding bulls raised in an extensive regimen. In addition, we propose a new real-time PCR assay targeting the *EF1-alpha-Tf1* gene, developed as an alternative method for the detection of *T. foetus* in bull preputial wash samples.

## Materials and methods

### Systematic review and selection of PCR protocols

**Fig. 1:**
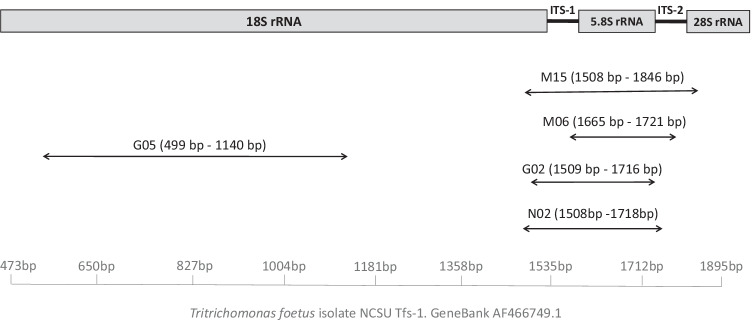
Primer position of the PCR protocols used that target the 18S/ITS-1/5.8S/ITS-2 genomic region of *T. foetus.* The reference and amplicon sequence length is indicated in base pair (bp). This figure is based on Fig. 1 from Felleisen et al. (1998) publication

We conducted a systematic bibliographic search in PubMed, search string: (Tritrichomonas foetus) AND (PCR)); Web of Science, search string: TS = (Tritrichomonas AND foetus AND PCR); and Scopus, search string: TITLE-ABS-KEY (“Tritrichomonas foetus”) AND (“PCR”). Among the papers found (*n* = 614), duplicates were removed using EndNote X8 software and the abstracts were manually screened. All publications that mentioned the use of a PCR technique for *T. foetus* detection (41 publications) were further evaluated according to the following inclusion criteria: (i) abstracts must contain information about the *T. foetus* genetic target, (ii) primers and probe sequences must be published, (iii) PCR and thermal cycler conditions must be provided, and (iv) due to the technological limitations of many diagnostic laboratories, the PCR design should be based on DNA detection (not RNA due its fragility in field samples) and, just in case of real-time PCR protocols, use the most common fluorescent systems (SYBRgreen or Taqman probes) for *T. foetus* identification. When the same primer set was used in more than one study, only one was included in the present study based on the following prioritization criteria: (i) those that were assessed on clinical samples and/or (ii) those adapted to real-time protocols, in particular, those using specific probes. Finally, six published protocols (five targeting the rRNA-ITS genomic region and one *TfRE*) were included in the study (Table 1). The locations of the selected primers within the rRNA-ITS region are shown in Fig. 1.

**Table 1:** PCR assays for *T. foetus* detection included in the present study

Target^a^	PCR type	Reference (code^b^)	Primers and probes (5′ to 3′)	PCR conditions^c^									
				Fw (µM)	Rv (µM)	Pr (µM)	ID. (C°/min)	D. (C°/s)	A. (C°/s)	E. (C°/s)	Nº	FE. (C°/min)	AS. (bp)
ITS	Real time (probe)	McMillen and Lewis 2006 (M06)	TFF2: GCGGCTGGATTAGCTTTCTTT	0.90	0.90	0.08	95/5^d^	95/20	60/45		40		57
			TFR2: GGCGCGCAATGTGCAT										
			TRICHP2: 6-FAM-ACAAG TTCGATCTTTG-MGB-BHQ										
ITS	Real time (SYBR)	Mueller et al. 2015 (M15)	TFR3: CGGGTCTTCCTATAT GAGACAGAACC	0.20	0.20		95/5	95/30	63/20		40		348
			TFR4: CCTGCCGTTGGATCAGTTTCGTTAA										
EF	Real time (probe)	Current study (V21)	Ftf_EF1A1: AGTCCGCCGCC AAATCAA	0.50	0.50	0.40	95/5	95/20	61/35		45		76
			Rtf_EF1A1: CTCTTCAACTTCGGCTGTGA										
			Ptf_EF1A1: 6-FAM- ATCATCAAGTACGGCTCAGT-MGB-NFQ										
ITS	Conventional (nested)^e^	Gookin et al. 2002 (G02)	TFR3: CGGGTCTTCCTATATGAGACAGAACC	1.25	1.25		95/15	95/30	57/30	72/30	50	72/10	347
			TFR4: CCTGCCGTTGGATCAGTTTCGTTAA										
			TFITS-F: CTGCCGTTGGATCAGTTTCG	2.50	2.50								208
			TFITS-R: GCAATGTGCATTCAAAGATCG										
ITS	Conventional	Gookin et al. 2005 (G05)	499F: GCTCGTAGTCAGAACTGC 1140R: CCCAATTAGAACTCT ATCTC	0.30	0.30		95/15	95/60	60.1/60	72/120	35	72/10	642
ITS	Conventional	Nickel et al. 2002 (N02)	TF211A: CCTGCCGTTGGATCAGTTTCGTTA	0.20	0.20		95/15	94/60	60/60	72/60	35	72/10	211
			TF211B: GCGCAATGTGCATTCAAAGATTCG										
TfRE	Conventional	Ho et al. 1994 (H94)	TF1: CATTATCCCAAATGGTATAAC	0.38	0.365		95/15	94/60	45/60	72/120	41	72/10	162
			TF2: GTCATTAAGTACATAAATTC										

^a^Genomic region that comprises the 18S, 5.8S, and 28S rRNA genes and the internal transcribed spacer 1 and 2 of *T. foetus* (ITS), *EF1-alpha-Tf1* gene (EF), and microsatellite *TfRE* sequence (TfRE)

^b^Internal code that references the protocols used in the current study

^c^PCR were performed in a 25 µL of final reaction volume for M06, V21, G02, G05, N02, and H94 protocols and 20 µL for M15 protocol: *Fw (µM)*, final concentration of forward primer; *Rv (µM)*, final concentration of reverse primer; *Pr (µM)*, final concentration of probe; *ID. (C°/min)*, initial denaturation; *D. (C°/s)*, denaturation step per cycle; *A. (C°/s)*, annealing step per cycle; *E. (C°/s)*, elongation step per cycle; *Nº*, number of cycles; *FE. (C°/min)*, final elongation step; *AS. (bp)*, amplicon size

^d^A previous step at 50 °C during 2 min was added

^e^It is a one-step nested PCR

All PCR protocols were carried out under the conditions of the original paper with minor changes for their adaptation to our laboratory (optimal conditions of the Qiagen PCR kits used: QuantiFast Pathogen PCR, QuantiFast SYBR Green PCR, and Qiagen Multiplex PCR kits). For real-time PCR, a probe specific for an internal PCR control was used (provided by the QuantiFast Pathogen PCR kit).

### Design and optimization of a novel real-time PCR based on the EF1-alpha-Tf1 gene

The design of an in-house PCR based on the *EF1-alpha-Tf1* gene (called V21, Table 1) was carried out using Oligo (Oligo 7 Primer Analysis Software) and HM217356.1 from the GeneBank database as the reference sequence. The specificity of the primers and probe were verified in silico by blastn using BLAST online software from the NCBI website (https://www.ncbi.nlm.nih.gov/). The primers were synthesized by Eurofins Genomics (Eurofins Genomics, GmbH) and the probe by ThermoFisher (Applied Biosystems, UK). The primer/probe sequences and PCR conditions are shown in Table 1. The QuantiFast Pathogen PCR kit (Qiagen, Germany) and an internal PCR control provided in the kit were used to verify the lack of PCR inhibition.

### Analytical sensitivity and specificity tests

Conventional PCR protocols were performed using C1000 and T100 thermocyclers (Bio-Rad, USA) and the PCR product detected following gel electrophoresis. For all conventional PCR assays, electrophoresis was performed in 2% agarose gels (Biotools MB agarose) at 70 mV and 400 mA for 55 min. Real-time PCR assays were performed in a CFX96 thermocycler (Bio-Rad, USA) using specific conditions (Table 1).

The validation data from the original studies (and subsequent studies), which assessed the specificity of each PCR assay, were taken into account for the analytical specificity test (Gookin et al. 2002, 2005; Nickel et al. 2002; McMillen and Lew 2006; Effinger et al. 2014; Mueller et al. 2015; Ginter Summarell et al. 2018; Dąbrowska et al. 2019a; Meggiolaro et al. 2019). In addition, information about the specificity of the genetic target on which the novel PCR protocol was based was considered (Oyhenart 2018). A panel of pathogens (field or reference strains) associated with bovine infertility or potentially present in preputial samples was also included (one strain/species). The field isolates were part of the VISAVET-UCM collection (Universidad Complutense de Madrid, Spain) and type strains came from the ATCC collection: *Arcobacter butzleri*, *Escherichia coli*, *Aeromonas hydrophila*, *Brucella abortus*, *Toxoplasma gondii*, *Salmonella enterica*, *Yersinia enterocolitica*, *Coxiella burnetii*, *Chlamydia abortus*, *Tritrichomonas foetus* (ATCC-30232 TM reference) *Campylobacter jejuni*, *Campylobacter coli*, *Campylobacter lanienae*, *Campylobacter sputorum*, *Campyobacter fetus*, *C. fetus* subsp. *venerealis*, *C. fetus* subsp. *fetus* (ATCC-27374 TM reference), and *C. fetus* subsp. *testudinum* (ATCC-BAA-2539 TM reference).

The ATCC-30232 strain was used as a control for the analytical sensitivity test of each evaluated PCR. The analysis was performed according to the OIE instructions (OIE 2019). The limit of detection (LOD) and corresponding confidence interval (CI) was calculated for each selected protocol using a battery of serial tenfold dilutions (triplicate test for real-time PCR and duplicate test for conventional PCR) starting from 1 ng of DNA/reaction (corresponding to approximately 5750 copies of the *T. foetus* genome (Benchimol et al. 2017)). The DNA concentration was adjusted using a High Sensitivity DNA Quantitation Kit (Invitrogen, USA) and a Qubit 4 Fluorometer device (Invitrogen, USA). For real-time protocols, the cut-off was established based on the arithmetic mean value of the *C*_T_ of the 20 replicates from the LOD test in which the highest dilution of the LOD had a CI ≥ 95% (OIE 2019). For conventional protocols, the LOD and CI were established according to the visual detection of bands in the agarose gel.

### Preputial washes and DNA extraction

A selection of 466 samples recovered for routine diagnosis from breeding bulls raised in extensive regimens was included. Samples were selected based on previous molecular results using an in-house PCR designed by Genetics PCR Solutions (GPS, Spain). The samples consisted of 15 mL of preputial washes in PBS, which were centrifuged at 1512 × *g* for 10 min and stored at − 80 °C until analysis. Nucleic-acid extraction was performed using the QIAamp DNA Mini Kit (Qiagen, Germany), with slight modifications: a cellular lysis buffer (20 mM Tris–Cl (pH 8), 2 mM sodium EDTA, 1.2% Triton X-100, and 20 mg/mL lysozyme in a 360 µL final volume) was first added to the sample pellets and the mixture incubated at 37 °C for 1 h. The DNA extraction was subsequently performed according to the manufacturer’s instructions.

### Clinical diagnosis classification of the results

In the current study, we used the M06 PCR protocol (McMillen and Lew 2006) as the reference assay, as previously reported (Meggiolaro et al. 2019). We considered non-specific results to be those for which *T. foetus*-positive results were obtained with only one PCR protocol (and negative with M06). The internal PCR control included in this protocol allowed us to verify the lack of inhibition.

The concordance between the results of each protocol and those of the M06 protocol was estimated with Cohen’s kappa coefficient using SPSS software (IBM SPSS Statistics 22.0) according to the following interpretation: 0.0–0.2: insignificant, 0.2–0.4: low, 0.4–0.6: moderate, 0.6–0.8: good, and 0.8–1.0: very good. The percentage of agreement of positive and negative samples of each protocol with the reference was calculated, along with the positive predictive value (PPV) and negative predictive value (NPV).

## Results

### In vitro analysis: analytical sensitivity and specificity tests

The analytical specificity test revealed no amplification with any control isolated strain for any PCR protocol (including the in-house V21 protocol), being only positive for the *T. foetus* isolate (ATCC-30232). The LOD (CI ≥ 95%) of the M06, M15, G02, G05, and H94 PCR protocols was 1.10^−4^ ng of DNA/reaction (approximately 0.575 T*. foetus* genome copies), whereas the N02 protocol had a LOD (CI ≥ 95%) of 1.10^−3^ ng of DNA/reaction (approximately 5.75 T*. foetus* genome copies). Further results related to the LOD of each PCR protocol are included in Table 2. After the analytical sensitivity assay, we established a cut-off for real-time protocols. The cut-off for the M06 protocol (here the reference assay) was a *C*_T_ ≤ 34 and that for the M15 protocol a *C*_T_ ≤ 32.

**Table 2:** Agreement results of the selected PCR protocols with respect to the M06 protocol results and percentage of non-specificities

PCR	Concordance of positives	Concordance of negatives	PPV	NPV	Non-specific results	LOD (ng/μL)	Cohen’s kappa	Genetic target	Reference
M06	Ref.*	Ref.*	Ref.*	Ref.*	0/466 (0%)	4.10^−6^	Ref.*	rRNA-ITS	McMillen and Lew 2006
M15	161/168 (95.8%)	296/298 (99.3%)	0.98	0.98	2/466 (0.4%)	5.10^−6^	0.958	rRNA-ITS	Mueller et al. 2015
G02	160/168 (95.2%)	209/298 (70.1%)	0.64	0.96	87/466 (18.6%)	4.10^−6^	0.587	rRNA-ITS	Gookin et al. 2002
G05	159/168 (94.6%)	296/298 (99.3%)	1	0.97	0/466 (0%)	4.10^−6^	0.948	rRNA-ITS	Gookin et al. 2005
N02	125/168 (74.4%)	295/298 (98.9%)	0.99	0.87	1/466 (0.2%)	4.10^−5^	0.700	rRNA-ITS	Nickel et al. 2002
H94	166/168 (98.8%)	296/298 (99.3%)	1	0.99	0/466 (0%)	4.10^−6^	0.986	*TfRE*	Ho et al. 1994
V21	161/168 (95.8%)	298/298 (100%)	1	0.98	0/466 (0%)	4.10^−5^	0.967	*EF1-alpha-Tf1*	Current study

See Table 1 to know all PCR references. From left to right in the header row is indicated: The PCR protocol (PCR); the agreement of positives samples with M06 (Concordance of positives) and the concordance of negatives where 168 are positive samples and 298 negative samples for M06 protocol; the positive predictive value (PPV) and the negative predictive value (NPV); the non-specific results (are those samples negative to the reference protocol and positive only by one of the others) where 466 are the total samples; the limit of detection (LOD) with a confidence interval ≥ 95%; the Cohen’s kappa value; the genetic target where it is indicate the genomic region that comprises the *18S*, *5.8S*, and *28S* rRNA genes and the internal transcribed spacer 1 and 2 (rRNA-ITS), TfRE microsatellite, and *EF1-alpha-Tf1* gene; and the reference of the original work

^*^The method used as reference (Ref.) was the M06 protocol

### Comparative study on field samples

A summary of the results on the field samples is shown in Table 2. The panel of included field samples was composed of 168 positive samples and 298 negative samples. The percentage of agreement among the PCR protocols targeting the rRNA-ITS region was variable. The results of M15 and G05 showed high agreement with those of M06 (95.8% and 94.6%, respectively, for positive samples and 99.3% for negative samples). The concordance of the results obtained with M15 with those of M06 was very good according to Cohen’s kappa value (0.958), and the PPV and NPV were 0.98 (Table 2). Similarly, Cohen’s kappa value for G05 was 0.948, with a PPV of 1 and a NPV of 0.97 (Table 2). Protocols G02 and N02 showed poorer results. The Cohen’s kappa value for the G02 protocol was 0.587 (moderate concordance), with 70.1% agreement for negative samples and a PPV of 0.64, whereas Cohen’s kappa value for the N02 protocol was 0.700 (good concordance), with 74.4% agreement for positive samples and a NPV of 0.87 (Table 2). The percentage of non-specific results for the rRNA-ITS—PCR protocols also varied; for the M15 protocol, only 2 of 466 total samples (0.4%) were falsely positive, 87 of 466 (18.6%) for the G02 protocol, and 1 of 466 (0.2%) for the N02 protocol.

The H94 protocol based on the microsatellite *TfRE* of *T. foetus* showed a Cohen’s kappa coefficient of 0.986 (very good concordance), with high agreement with the M06 protocol for positive and negative samples: PPV = 1 and NPV = 0.99 (Table 2).

### In-house PCR protocol based on the EF1-alpha-Tf1 gene

The analytical sensitivity test showed a LOD (CI ≥ 95%) of 1.10^−3^ ng of DNA/reaction (approximately 5.75 T*. foetus* genome copies) and the cut-off was established at a *C*_T_ ≤ 40. In terms of the performance of V21 on field samples, this PCR assay showed a Cohen’s kappa value of 0.967 (very good concordance), with 95.8% concordance for positive samples and 100% for negative samples with our reference PCR (M06 protocol) and a PPV of 1 and NPV of 0.98 (Table 2). These results are in accordance with the analytical specificity results of the V21 protocol, which showed no amplification with the isolates included in the specificity test (except *T. foetus* isolates, Table 2).

## Discussion

*T. foetus* is the etiological agent of trichomonosis, which is generally an asymptomatic and persistent venereal disease in bulls, whereas it can cause a number of reproductive disorders in cows (Michi et al. 2016). In addition, this parasite can be transmitted through natural breeding and artificial insemination (Givens 2018). In this scenario, the detection of *T. foetus* in males is an essential step for the control of trichomonosis. The OIE thus established real-time PCR as a recommended technique for the detection of *T. foetus* in clinical samples (OIE 2018). The OIE guidelines of trichomonosis diagnosis include the PCR protocol of McMillen and Lew (McMillen and Lew 2006) as a suitable assay for *T. foetus* identification in clinical samples. Here, we used this protocol (called M06) as the reference method (as previously considered by Meggiolaro et al. 2019) to compare five different PCR protocols currently available for *T. foetus* detection (Table 1). This is the first report of a systematic comparison of PCR techniques available for *T. foetus* detection directly on bull preputial samples. Five protocols based on rRNA-ITS were selected (Table 1): (1) M06 (McMillen and Lew 2006) (the reference assay in our study), (2) M15 (Mueller et al. 2015), (3) G02 (Gookin et al. 2002), (4) G05 (Gookin et al. 2005), and (5) N02 (Nickel et al. 2002). This genomic region is a multicopy target (Chakrabarti et al. 1992) that appears to be highly similar in other *Tritrichomonas* species, such as *Tritrichomonas mobilensis*, resulting in potential cross-reactions, as previously reported for primers TFR3/TFR4 (M15 protocol) (Dąbrowska et al. 2019a).

Although *T. mobilensis* has only been described in the intestine of *Saimiri sciureus* and *Saimiri boliviensis* squirrel monkeys (Scimeca et al. 1989) to date, potential target homology suggests a potential cross-reaction with other *Tritrichomonas* spp., which could pose a problem in the analysis of field samples. According to our results, G02 (TFIT-F/TFITS-R set primers) showed cross-reactions with *Simplicimonas* spp. by in silico analysis of sequences from PCR amplicons sequencing (data not shown). In a previous work by Frey et al. (2017), similar cross-reactions were noted in vaginal swabs of cows and heifers with other set of primers and probes. This data reinforces the idea that PCR protocols based on rRNA-ITS region may provide false positives (Frey et al. 2017; Dąbrowska et al. 2019a). Thus, PCR techniques based on alternative targets could become a useful approach to detect *T. foetus* as a confirmatory test.

In terms of alternative genetic targets for the detection of *T. foetus* directly on clinical samples, one PCR protocol based on the microsatellite *TfRE* is available: H94 (Ho et al. 1994). This protocol was designed based on a barely studied repetitive region of the *T. foetus* genome, with a single available reference sequence in GenBank (AY435432.1). Finally, according to the OIE (2018), a commercial PCR kit targeting the *beta-tubulin 1* gene is also available. However, the information to perform the assay is not published, which is why it was not included in our comparative study. As a consequence, the number of alternatives to rRNA-ITS-based PCR protocols is very limited. Recently, a LAMP technique targeting the *EF1-alpha-Tf1* gene showed very good results for *T. foetus* identification in bull preputial samples, being able to detect 0.5 trophozoites/mL (Oyhenart 2018). However, no PCR protocol based on this target has been published. We therefore propose a novel real-time PCR assay (here called the V21 protocol) targeting the *EF1-alpha-Tf1* gene as an alternative target for the confirmation of the presence of *T. foetus* in preputial samples (Table 1).

In terms of the protocols targeting the rRNA-ITS genomic region, our results from the comparative study showed very good concordance for the M15 protocol, for which Cohen’s kappa value was 0.958, and the G05 protocol, for which Cohen’s kappa value was 0.948 (Table 2). The M15 protocol is a real-time adaptation (Mueller et al. 2015) of the conventional PCR protocol from the study of Felleisen et al. (Felleisen et al. 1998). Thus, their primers TFR3/TFR4 (Table 1) have been evaluated and used by several studies for *T. foetus* identification (Casteriano et al. 2016; Dąbrowska et al. 2019a), with satisfactory results. On the contrary, it is difficult to find publications that have used the primer pair 499F/1140R (protocol G05, Table 1) (Tolbert et al. 2012). However, we found the diagnostic performance of the G05 protocol to be very similar to that of the M15 and M06 protocols. Thus, the G05 protocol appears to be relatively good for the detection of *T. foetus* in bull preputial wash samples.

On the other hand, Cohen’s kappa value for the G02 protocol was 0.587 (PPV = 0.6), with 18.6% non-specific results. These results suggest the presence of a high number of false positives, whereas for the N02 protocol, Cohen’s kappa value was 0.700, with a NPV of 0.87, suggesting the presence of false negatives (Table 2). Our results differ from those of the original publications of both protocols (Gookin et al. 2002; Nickel et al. 2002). The absence of additional diagnostic studies using these protocols hampers a proper discussion of this point. Nevertheless, according our results, G02 PCR is a limited technique for the identification of *T. foetus* directly on clinical samples (PPV = 0.64, Table 2). Further studies will be needed to confirm the diagnostic performance of the G02 and N02 protocols.

The H94 protocol, based on the microsatellite *TfRE*, showed a Cohen’s kappa coefficient of 0.986, with no observed non-specific amplification (Table 2), contradicting the results cited in the study of Felleisen et al. (Felleisen et al. 1998). Nevertheless, our results are similar to those of the study of Ho et al. (Ho et al. 1994) and Riley et al. (Riley et al. 1995), in which the use of the primer pair TF1/TF2 appeared to provide high performance for the detection of *T. foetus*. Our study shows that the diagnostic performance of the H94 protocol on field samples appears to be good, with a PPV of 1 and NPV of 0.99.

Finally, Cohen’s kappa coefficient for the in-house V21 protocol (based on the *EF1-alpha-Tf1* gene) was 0.967 (with a PPV of 1 and NPV of 0.98), and there was no non-specific amplification, as for the H94 protocol (Table 2). Thus, V21 protocol seems to be highly specific compared with other PCR designs based on rRNA-ITS genomic region (Table 2) (Frey et al. 2017; Dąbrowska et al. 2019a). Nevertheless, the LOD of the V21 protocol should be considered, as it was an order of magnitude lower than that of M06 (the reference protocol), detecting approximately 5.75 T*. foetus* genome copies per reaction. Based on our results, the use of the H94 and V21 protocols appears to be a good approach for the confirmation of clinical cases when alternative genomic regions to rRNA-ITS are required. An important aspect of the V21 protocol relative to the H94 protocol, for *T. foetus* detection on clinical samples, is the use of an internal PCR control to detect inhibition, the absence of non-specificities, and the rapid obtention of results, as it is a real-time PCR assay with a specific probe.

## Conclusions

Published PCR protocols based on the rRNA-ITS region showed a high degree of agreement (M15 and G05) with the reference protocol (M06) to identify *T. foetus* in preputial bull samples. However, the rRNA-ITS genomic region appears to be highly similar in phylogenetically close species, which may translate into potential cross-reactions. Thus, protocols based on alternative molecular targets may be useful. The H94 protocol based on the microsatellite *TfRE* (AY435432.1 GeneBank) showed excellent concordance with the reference protocol (Cohen’s kappa coefficient of 0.986), with no non-specific amplification detected. Our study shows the diagnostic performance of H94 in preputial bull samples to be good, capable of detecting approximately 0.575 copies of *T. foetus* genome per PCR reaction.

Our in-house V21 protocol based on the *EF1-alpha-Tf1* gene also showed very high concordance with the reference protocol (Cohen’s kappa coefficient of 0.967), with no non-specific amplification, to detect 5.75 copies of *T. foetus* genome per PCR reaction. Both the H94 and V21 protocols show promise for confirming clinical cases of *T. foetus* when another molecular target is required. An advantage of the V21 over the H94 protocol, for *T. foetus* detection on clinical samples, is the use of an internal PCR control to detect inhibition and the speed to obtain results as it is a specific probe real-time PCR.
